# High intensity, short duration pulling in heavy horses: physiological effects of competition and rapid weight change

**DOI:** 10.1186/s12917-017-1243-9

**Published:** 2017-11-07

**Authors:** Persephone Greco-Otto, Shannon Massie, Erin Shields, Marie-France Roy, Edmond Pajor, Renaud Léguillette

**Affiliations:** Department of Veterinary Clinical and Diagnostic Sciences, Faculty of Veterinary Medicine, University of Calgary, Calgary, AB T2N 4N1 Canada

**Keywords:** Draft, Equine, Rapid weight loss, Cardiac troponin, Electrolytes, Serum chemistry

## Abstract

**Background:**

The Heavy Horse Pull is a competition where teams of two horses pull an increasingly heavy sled for a short distance. Similar to human wrestlers, some horses may undergo rapid weight change in order to enter a lower weight category. The objectives were to study the physiological effects of this practice as well as of the pulling competition in draft horses.

**Results:**

Fifty horses were divided into light-, middle- and heavyweight categories based on their arrival weights and competed 1–3 days after. Body weight was measured upon arrival and pre-competition. Blood was sampled for chemistry and high sensitivity cardiac troponin T (hscTnT) at arrival, pre- and post-competition in 34, 26 and 20 horses, respectively. Body weight increased significantly between arrival and pre-competition for light (7.2% (Median: 62.8Kg (41.7–77.0)) and middle (8.6% (Median: 80.5Kg (62.7–90.9)) weight categories. Change in body weight was correlated (*r* = 0.69, *p* = 0.002) with competition ranking for middleweights. The ratios of weight pulled to team body weight were 2.7 (1.9–2.8), 2.6 (2.5–2.6) and 2.4 (2.2–2.5) for the lightweights, middleweights and heavyweights, respectively. Blood chemistry indicated hemoconcentration on arrival in the middleweight and lightweight horses. Hemoconcentration was not seen on arrival in some horses with marked rapid weight change. Overall, no chemistry parameter changed between pre- and post-competition. The hscTnT stayed within normal range post-competition.

**Conclusions:**

While horses arrived to the event with indications of hemoconcentration, they appeared to have sufficient time to rehydrate prior to competition, and the effects of the competition were reversible within 3 h.

## Background

The Heavy Horse Pull is a competition that showcases horses’ strength and anaerobic power. Teams of two draft horses pull a weighted sled over 14 ft at increasing load until a maximum is reached. Similar to many human sports, horses compete within a specified weight division and undergo mandatory weigh-ins to ensure equal and fair competition. In wrestling, human athletes attempt to gain what is believed to be a competitive advantage by weighing in at the top of a lower weight division and commonly drop 3–4 kg in the week prior to competition [1]. Weight changes in human athletes are primarily attributed to total body water loss and often exceed 2% of body weight [2]. This rapid weight loss (RWL) is achieved by severely restricting fluid and caloric intake, using laxatives and increasing perspiration [3]. Some heavy horses may also undergo similar RWL practices, commonly referred to as “shrinkage”, but unlike human wrestlers, they have 1 to 3 days after weigh-in to rehydrate and regain weight prior to competition. This is a key difference, as these horses undergo rapid weight changes (weight loss followed by weight gain), rather than simply rapid weight loss. Though not documented in the scientific literature, shrinkage is common enough that some pulling competitions explicitly state that excessive shrinking is grounds for disqualification. While RWL in humans has negative physiological effects on the renal, muscular and cardiac systems [4, 5], the effects of severe short term changes in weight are unknown in horses.

Additionally, the physiological response to short term high intensity resistance pulling in heavy horses is unknown. Previous work examined the physiological effects of sustained pulling in Draft horses [6, 7], however the workload efforts, physiological demand and metabolic stress of the Heavy Horse Pull are drastically different since it is at a much higher intensity, but for a much shorter duration. The effects of a high intensity short duration exercise on the myocardium are unknown in horses but have been shown to induce a leakage of cardiac troponin in human athletes [8]. In addition, we reported that high sensitivity cardiac troponin (hscTnT) can be increased in racehorses with upper airway obstruction while racing [9]. This may be due to myocardium damage or increased permeability of myocardial membranes or release of cardiac troponin through vesicles [10–12]. It has been documented that 35% of large horse breeds competing in hitch and pulling classes have recurrent laryngeal neuropathy [13], which can result in a drastic reduction in cross-sectional area of the upper airway. Consequently, we hypothesized that hscTnT increases in draft horses competing in pulling events.

The aim of the study was to assess the effect of competition, including “shrinkage”, on the physiology of draft horses. We hypothesized that rapid weight changes happen in the lightweight and middleweight categories, which affects performance and results in hemoconcentration and electrolytes imbalances. Additionally, we hypothesized that competition itself would effect plasma homeostasis and induce subclinical myocardial damage. The specific objectives of the study were therefore: 1) To quantify the extent and prevalence of rapid weight changes in this competition and its effect on physiology and performance; and 2) To asses the physiological effects of the Heavy Horse Pull competition on blood chemistry, electrolytes, and hscTnT.

## Methods

### Heavy Horse Pull competition

The study took place during the official Heavy Horse Pull competition at the 2015 Calgary Stampede. For logistical reasons, Calgary Stampede divides teams into three equal weight categories based on entry weight (lightweight: <1451Kg, middleweight: 1452-1587Kg, heavyweight: >1587Kg). Horses are weighed on arrival (day 1). Lightweights compete on day 2, middleweights on day 3 and heavyweights on day 4, as per Calgary Stampede regulations.

Competition results including rank, maximal weight and distance pulled were collected and analyzed for all horses that entered the competition. Additionally, historical data (over the last 13 years) of the Calgary Stampede heavy horses pulling competition maximal weight pulled results for each weight category were available for discussion.

### Horses

Body weight, competition results, horses’ age, hauling distance, last feeding and last water prior to arrival was available from the organizers for all 52 horses participating in the event. Out of the 52 horses competing, 35 draft horses (*n* = 18 teams) were voluntarily enrolled in the study for blood data collection for which owners completed a consent form. This study was approved by the University of Calgary Veterinary Sciences Animal Care Committee.

### Physical exam

All horses underwent and passed the Calgary Stampede official “Fitness to Compete” veterinary check, which included a lameness exam, cardiac auscultation, checking for upper airway noise, and heart rate recovery.

### Body weight measurements

To quantify the extent and prevalence of rapid weight change (specific objective 1), all horses (*n* = 52) were weighed on arrival and prior to competition. Indeed, the weight gained between arrival and pre-competition is assumed to be indicative of the weight lost during the “shrinkage” process done prior to arrival. This is because “shrinkage” is believed to create a competitive advantage, where owners want the horses to weigh in at lower-than-normal body weights, but induce rapid weight change (gain) to return to a higher body weight prior to competition. Although documenting the normal body weight on farm would have allowed using it as a reference, it was not part of the specific objectives of the study and it was not practical to do as horses came from across North America.

All horses (*n* = 52) were weighed between 6 am and 7 am on arrival (day 1) and were assigned to a weight category on that day as per official Calgary Stampede rules and regulations. All horses, except for one pair (*n* = 50 total), were again weighed on the morning prior to competition (day 2, 3 and 4 for lightweight, middleweight and heavyweight, respectively) for the purpose of the study, though this had no effect on weight division designation. Horses were not weighed following competition, since it was outside the scope of the study to document “shrinkage.” In addition, it was not practical, as horses had to leave the premises following competition. The scale was calibrated 41 days prior to the study.

### Blood sampling

Venous blood samples were drawn in 7 mL lithium heparin tubes at 3 time points: On arrival (day 1), on the morning of the competition (pre-competition, day 2 for lightweights, day 3 for middleweights and day 4 for heavyweights) and 3 h after the competition (post-competition). Samples were immediately centrifuged (10 min at 2000 x *g*) and plasma was immediately frozen on site at −20 °C, then at -80 °C until they underwent chemistry,^1^ electrolyte^1^ (commercial veterinary laboratory) and hscTnT analysis^2^^,^ 3 ^,^ 4 (commercial human hospital laboratory) within the next month. Chemistry and electrolytes analysis included total protein, albumin, globulins, urea, creatinine, calculated osmolarity (equation: 2 * (Na + K)) + (BUN/2.8) + (Glucose/18)), sodium, chloride, potassium, creatinine kinase (CK), aspartate aminotransferase (AST), alanine aminotransferase (ALT), glucose, total bilirubin. The plasma analysis results were grouped as total protein, albumin, creatinine and urea and calculated osmolarity to study hemoconcentration. Sodium, chloride and potassium were grouped to study specifically electrolytes data. The CK and AST were described together to study muscle damage. The cTnT was used as a specific indicator of myocardial damage. Lastly, glucose, ALT and total bilirubin were reported as other chemistry parameters. Out of the 35 horses for which consent was obtained for blood collection, 34 were sampled on arrival (1 horse too difficult to sample on arrival), 26 were sampled pre- and 20 were sampled post-competition (samples missing due to horses being withdrawn from the study, owner compliance or logistical reasons).

### Statistical analysis

Body weight difference between arrival and pre-competition was tested using a paired t-test for all horses (*n* = 50) and a Wilcoxon matched-pairs signed rank test for within-each category (light, middle and heavy) comparison. Differences between the 3 weight categories in maximum weight pulled and weight ratios was tested using a Kruskal Wallis test with a Dunn’s post hoc test for multiple comparisons.

Differences in blood chemistry parameters and electrolytes between arrival, pre-competition and post-competition were analyzed using a repeated ANOVA, followed by a Tukey post hoc test (data from all horses together) or a Kruskal Wallis test (for changes within each weight category), with a Dunn’s post hoc test for multiple comparisons. The exception was for the lightweight category where only arrival and pre-competition samples were studied for differences in blood parameters using a Wilcoxon signed rank test because only 3 horses were sampled post-competition and this group was not included in the analysis. Association between weight change and final rank as well as between chemistry parameters from arrival samples were examined using a Pearson Correlation (data from all horses together) or a Spearman Correlation (for horses within each weight category). Data is presented as median and interquartile ranges to accommodate non-normal distribution of data (similar format was applied to the data involving all of the horses for consistency).

## Results

### Horses

The information provided by the owners to the event’s staff indicated that horses were 9 years old (IQR: 8–10), were hauled a median distance of 451Km (IQR: 100–800)) and were fed 10 h (IQR: 2–11)) prior to weigh-in. No horses were disqualified based on the “Fitness to Compete” veterinary exam.

### Body weight changes

Arrival (day 1) and pre-competition (day 2, 3 or 4 depending on division) weight was recorded for 50 out of 52 horses competing in the Heavy Horse Pull competition (1 team of 2 horses in the lightweight division missed pre-competition weighing).

**Fig. 1:**
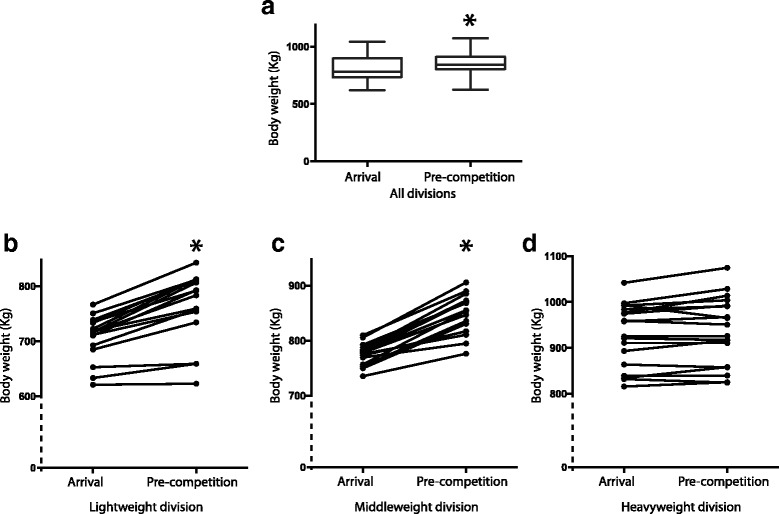
Body weight change between arrival and pre-competition in draft horses participating in the Heavy Horse Pull competition at the Calgary Stampede. **a** Body weight (in Kg) measured at arrival and pre-competition in all 50 horses participating in the event. **b** Body weight change in the 16 horses from the lightweight division between arrival and pre-competition. **c** Body weight change in the 16 horses from the middleweight division between arrival and pre-competition. **d** Body weight change in the 18 horses from the heavyweight division between arrival and pre-competition. * indicates significantly different from arrival

Overall for all horses, body weight increased significantly between the arrival (median: 779.0Kg (735.3–885.8)) and pre-competition (median: 844.8Kg (807.3–909.2)) weigh-in (*p* < 0.0001) (Fig. 1).

When looking at specific weight divisions, body weight increased by 7.2% (median: 62.8Kg (41.7–77.0)) (*p* < 0.0001) between arrival and pre-competition (day 2) for horses competing in the lightweight division (*n* = 16; 8 teams) (Fig. 1). Based on the competition weigh-in thresholds, 6 of the 8 lightweight teams would have qualified for the middleweight division when using the pre-competition weights. Similarly, body weight increased by 8.6% (median: 80.5Kg (62.7–90.9)) (*p* < 0.0001) between arrival and pre-competition (day 3) for horses competing in the middleweight division (n = 16; 8 teams) (Fig. 1). As a result, 7 of the 8 teams exceeded the middleweight division and would have qualified for the heavyweight division. Weight change was not significant in the heavyweight division (*n* = 18; 9 teams) (*p* = 0.053) (Fig. 1).

### Weight and ratio of weight pulled

**Fig. 2:**
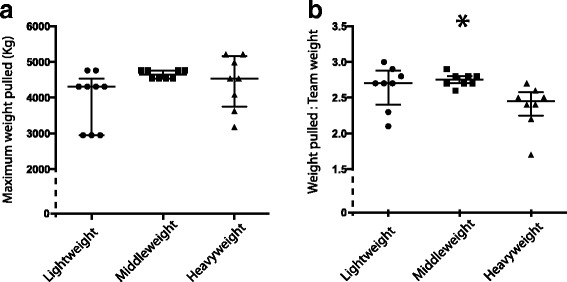
Maximum weight pulled and weight ratios: **a** Maximum weight pulled (in Kg) by 24 draft horse teams (48 horses total – 1 team was disqualified before reaching maximum weight). Horses are divided based on weight division. **b** Ratio of weight pulled to team weight (Kg) for 24 draft horse teams (48 horses total – 1 team was disqualified before reaching maximum weight). Horses are treated as teams (pairs) with combined total body weights. * indicates significantly different from heavyweights

The maximal weight pulled for each team is presented in Fig. 2. Ranking is determined by maximum weight and distance pulled (if total distance of 14 ft is not completed). For this reason, there are multiple teams that pulled the same maximal weight, though for different distances. The maximum weight pulled during the competition was 5216Kg by a heavyweight team, though this was not significantly greater than the maximal weight pulled by the winning middleweight or lightweight teams.

Additionally, the middleweights pulled a greater ratio of weight (weight pulled: team weight) than the heavyweights (median: 2.6 (2.5–2.6); 2.4 (2.2–2.5) respectively) (*p* = 0.01) (Fig. 2). There was no difference in weight pulled: team weight ratio between the lightweights (median: 2.7 (1.9–2.8)) and middleweights or lightweights and heavyweights.

### Weight change vs. competition rank

**Fig. 3:**
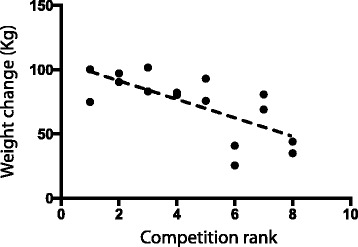
Correlation between weight change (between arrival and pre-competition, in Kg) and rank for 16 horses competing in the middleweight division. A lower rank is indicative of a better final placing

A strong negative correlation (*r* = −0.69, *p* = 0.002) was found in the middleweight horses between weight change and rank when horses were individually analyzed (Fig. 3). A higher rank is reflected with a lower placing (i.e. 1st place) therefore resulting in a negative correlation where the more weight change the better the competition result. Such correlation was not found for the lightweight and heavyweight divisions.

### Plasma sample analysis

Plasma samples were collected upon arrival from all horses volunteering for the study (*n* = 35, minus 1 horse that was too difficult to sample on arrival: lightweight *n* = 8, middleweight *n* = 12, heavyweight *n* = 14). Eight horses (23%) were not sampled at further time points in the study because owners were not present or withdrew from the study. Therefore 26 (74%) pre-competition samples were collected on days 2 to 4 (lightweight *n* = 7, middleweight n = 8, heavyweight *n* = 11). Twenty (57%) post-competition samples were obtained (lightweights n = 3, middleweights n = 8, heavyweights *n* = 9) as some teams had to leave the premises prior to sampling. Median and interquartile range for all plasma parameters at arrival, pre- and post-competition are provided in Table 1 for all horses that participated in the study. Correlations between plasma parameters for all horses are provided in Table 2. Within weight categories, correlations were only found in the lightweight division.

**Table 1:** Plasma chemistry, electrolytes and high sensitivity cardiac troponin in heavy horse pulling competition: Samples on arrival (day 1), on the morning of competition (day 2, 3 or 4, depending on weight category), and 3 h after competition (number of horses indicated for each time point). The first column is based on all horses, with the other columns divided based on weight category. Median (Interquartile Range) shown

	All			Lightweight			Middleweight			Heavyweight		
	Arrival	Pre	Post	Arrival	Pre	Post	Arrival	Pre	Post	Arrival	Pre	Post
N =	34	26	20	8	7	3	12	8	8	14	11	9
Total protein (g/L)	76.5 (71.3–81.3)	70.0 ^a^ (68.3–72.8) (*p* < 0.001)	70.5 ^a^ (66.0–74.0) (*p* < 0.001)	76.0 (72.0–80.3)	71.0 (69.5–72.5)	71.0 (69.5–73.5)	78.5 (70.8–90.0)	71.0 (70.0–72.8)	71.0 (68.0–74.3)	75.0 (69.0–79.0)	68.0 (62.0–72.0)	67.0 (66.0–73.0)
Albumin (g/L)	32.5 (31.0–36.0)	29.5 ^a^ (28.0–31.0) (*p* < 0.001)	30.0 ^a^ (28.0–32.0) (*p* < 0.001)	31.5 (30.0–32.5)	29.0 ^a^ (29.0–30.0) (*p* = 0.02)	30.0 (29.0–31.0)	37.5 (32.5–39.0)	31.5 ^a^ (27.8–33.5) (*p* = 0.003)	30.0 (29.0–34.3)	32.0 (32.0–34.0)	28.0 ^a^ (26.5–30.0) (*p* = 0.003)	30.0 (26.0–32.0)
Globulin (g/L)	43.0 (39.3–46.8)	41.0 (39.0–43.0)	40.5 (37.0–42.0)	46.0 (42.5–47.8)	42.0 (40.0–43.0)	41.0 (40.5–42.5)	41.5 (39.8–51.0)	41.5 (38.8–43.5)	40.5 (39.0–41.3)	42.5 (39.0–45.0)	40.0 (37.0–43.0)	38.0 (37.0–42.0)
Creatinine (mmol/L)	121.0 (102.8–144.0)	105.5 ^a^ (92.5–111.75) (*p* = 0.001)	111.0 ^a^ (89.8–120.3) (*p* = 0.001)	121.0 (113.0–155.0)	111.0 ^a^ (105.0–112.0) (*p* = 0.015)	115.0 (113.0–118.0)	124.5 (118.0–179.3)	106.0 ^a^ (96.0–110.0) (*p* = 0.005)	107.0 (88.5–120.0)	109.5 (98.5–129.8)	94.0 (90.5–117.0)	100.0 (90.0–121.0)
Urea (mmol/L)	5.9 (5.2–6.9)	4.6 ^a^ (4.2–5.7) (*p* < 0.001)	4.8 ^a^ (4.2–5.7) (*p* < 0.001)	6.6 (6.4–6.9)	5.8 ^a^ (5.6–6.5) (*p* = 0.047)	5.5 (5.2–6.5)	6.7 (5.9–10.5)	4.2 ^a^ (3.7–5.0) (*p* = 0.004)	4.0 (3.4–5.2)	5.1 (4.9–5.7)	4.6 (4.5–4.9)	5.4 (4.4–5.6)
Osmolarity (mmol/Kg)	267.0 (258.3–278.5)	256.0 ^a^ (245.3–262.8) (*p* < 0.001)	250.5 ^a^ (241.5–259.5) (*p* < 0.001)	267.5 (257.8–274.8)	261.0 (259.5–263.5)	258.0 (254.5–263.5)	281.0 (256.5–295.0)	257.5 (239.5–263.3)	242.0 ^a^ (241.0–247.5) (*p* = 0.006)	267.0 (264.3–267.8)	247.0 ^a^ (238.0–256.0) (*p* = 0.013)	250.0 (243.0–265.0)
Sodium (mmol/L)	135.0 (128.5–138.8)	129.5 ^a^ (123.3–131.0) (*p* < 0.001)	125.5 ^a^ (122.0–131.5) (*p* < 0.001)	134.0 (129.3–137.0)	130.0 (130.0–132.0)	131.0 (129.0–133.0)	140.0 (127.5–144.0)	129.0 (120.5–131.8)	123.0 ^a^ (121.8–126.0) (*p* = 0.02)	134.0 (133.0–135.0)	125.0 ^a^ (119.5–129.5) (*p* = 0.02)	125.0 (122.0–133.0)
Chloride (mmol/L)	99.0 (96.0–103.0)	95.0 ^a^ (86.0–98.0) (*p* = 0.004)	92.0 ^a^ (82.8–96.0) (*p* = 0.004)	98.0 (90.0–102.0)	96.0 (93.0–97.0)	91.0 (86.0–95.0)	102.0 (95.5–105.3)	92.5 (83.0–99.5)	91.0 ^a^ (82.3–94.3) (*p* = 0.01)	98.0 (97.0–101.0)	94.0 (89.0–98.0)	93.0 (88.0–101.0)
Potassium (mmol/L)	3.4 (3.2–3.7)	3.3 (2.8–3.7)	2.9 ^a^ (2.4–3.4) (*p* = 0.034)	3.45 (3.2–3.7)	3.6 (3.1–3.7)	2.3 (2.1–2.6)	3.3 (3.2–3.5)	3.3 (2.9–4)	2.8 ^a^ (2.2–2.8) (p = 0.01)	3.3 (3.1–3.6)	3.2 (2.5–3.5)	3.3 (3.1–3.5)
CK (IU/L)	175.5 (150.3–253.0)	174.0 (127.8–325.3)	225.5 (142.0–887.5) ^a^ (*p* = 0.04)	214.0 (189.0–313.8)	215.0 (169.5–622.5)	294.0 (230.0–704.0)	175.0 (160.8–259.0)	143.5 (113.3–504.0)	209.0 (147.5–753.5)	167.0 (147.3–213.8)	178.0 (115.5–293.5)	231.0 (142.0–4544.0)
ALT (IU/L)	7.0 (6.0–15.0)	6.5 (5.3–14.0)	9.5 (6.8–23.5)	6.0 (5.8–6.5)	7.0 (6.0–9.5)	7.5 (7.3–7.8)	11.5 (6.5–21.5)	14.0 (9.0–22.0)	14.5 (10.0–23.5)	7.0 (6.0–9.0)	5.0 (3.5–6.5)	6.0 (5.0–13.0)
cTnT (ng/L)	2.9 (2.9–6.0)	2.9 (2.9–5.5)	4.0 (2.9–10.0)	2.9 (2.9–5.1)	2.9 (2.9–11.5)	15 (8.9–16.5)	4.0 (2.9–7.0)	2.9 (2.9–5.7)	3.5 (2.9–9.5)	2.9 (2.9–3.8)	2.9 (2.9–5.0)	4.0 (2.9–8.0)
Glucose (mmol/L)	5.9 (5.4–6.6)	5.4 ^a^ (5.0–5.6) (*p* = 0.026)	5.5 (5.1–5.8)	5.8 (5.1–6.1)	5.4 (5.2–5.7)	5.3 (5.2–5.4)	6.4 (5.9–7.2)	5.4 ^a^ (5.2–5.8) (*p* = 0.047)	5.8 (5.4–6.4)	5.8 (5.3–6.1)	5.0 ^a^ (4.8–5.5) (*p* = 0.044)	5.5 (5.0–5.7)
T. bilirubin (μmol/L)	26.4 (19.9–31.3)	17.7 ^a^ (14.9–21.8) (*p* = 0.01)	17.8 (16.3–24.8)	25.0 (16.4–30.0)	15.0 ^a^ (12.9–18.8) (*p* = 0.01)	19.3 (18.3–23.6)	48.3 (28.7–62.3)	18.7 ^a^ (17.2–37.4) (*p* = 0.02)	18.4 (16.3–32.1)	21.8 (19.3–25.9)	17.5 (15.3–21.2)	17.3 (13.1–21.1)

^a^ significantly different from arrival. *CK* Creatinine Kinase, *hscTnT* High sensitivity cardiac troponin T

**Table 2:** Significant correlations between plasma chemistry parameters for horses sampled at the Heavy Horse Pull (*n* = 34) on arrival (day 1). Correlation is organized in descending order with the associated *p*-value

Blood parameter	Correlation coefficient	*P*
Globulin/Total protein	0.959	< 0.001
Creatinine/Urea	0.945	< 0.001
Total bilirubin/Urea	0.942	< 0.001
ALT/AST	0.93	< 0.001
Albumin/Sodium	0.917	< 0.001
Total bilirubin/Creatinine	0.905	< 0.001
Albumin/Total protein	0.896	< 0.001
ALT/CK	0.882	< 0.001
Total protein/Sodium	0.874	< 0.001
Total protein/Urea	0.839	< 0.001
Albumin/Urea	0.826	< 0.001
Glucose/Urea	0.819	< 0.001
Albumin/Total bilirubin	0.814	< 0.001
Glucose/Total bilirubin	0.808	< 0.001
Glucose/Total protein	0.788	< 0.001
Glucose/Creatinine	0.777	< 0.001
Glucose/Albumin	0.767	< 0.001
Total protein/Creatinine	0.759	< 0.001
Globulin/Urea	0.758	< 0.001
Sodium/Urea	0.753	< 0.001

No differences were found between pre- and post- competition for any of the blood parameters analyzed.

#### Total protein, albumin, creatinine, urea and calculated osmolarity

Overall, total protein was significantly greater at arrival (median: 76.5 g/L (71.3–81.3)) than at pre- (median: 70.0 g/L (68.3–72.8)) or post-competition (median: 70.5 g/L (66.0–74.0)) (*p* < 0.001) (Table 1). On arrival, 26 horses (76%) had a total protein value above 70 g/L, with a maximal value of 92 g/L observed in one horse. Analyzing the change in total protein between pre- and post-competition, we found that out of the 19 horses for which both a pre- and post-competition sample was available, 9/19 horses had a decrease in total protein after competition, whereas 10/19 horses had an increase in total protein (overall change in total protein pre- vs post-competition: Median: 0 g/L (−3.5, 3.5)). The changes in total protein were not significant when horses were split by weight categories.

Similarly to total protein, albumin was overall significantly greater at arrival (32.5 g/L (31.0–36.0)) than at the pre- (29.5 g/L (28.0–31.0)) or post-competition (30.0 g/L (28.0–32.0), respectively) time points (*p* < 0.001). Analysis of the data by weight categories showed that albumin decreased significantly between arrival (median: 37.5 g/L (32.5–39.0); (32.0 g/L (32.0–34.0), respectively) and pre-competition (median: 31.5 g/L (27.8–33.5); 28.0 g/L (26.5–30.0), respectively) for the middle and heavyweight horses (*p* = 0.02 and *p* = 0.003 respectively).

Creatinine was overall significantly greater on arrival (median: 121.0 mmol/L (102.8–144.0)) than at pre- (median: 105.5 mmol/L (92.5–111.75)) and post-competition (median: 111.0 mmol/L (89.8–120.3)) time points (*p* = 0.001 for both). Similarly, creatinine was greater on arrival than at pre-competition for the lightweight and middleweight divisions (*p* = 0.015 and *p* = 0.005 respectively) (Table 1). Five horses (15%) had elevated creatinine levels (Normal range: 70-159 mmol/L) on arrival, reaching a maximum of 235 mmol/L in one horse.

Urea overall significantly decreased from arrival (median: 5.9 mmol/L (5.2–6.9)) to pre- (median: 4.6 mmol/L (4.2–5.7)) and post-competition (median: 4.8 mmol/L (4.2–5.7)) (*p* < 0.001 for both). Urea was greater on arrival (median: 6.6 mmol/L (6.4–6.9); 6.7 mmol/L (5.9–10.5), respectively) than pre-competition (median: 5.8 mmol/L (5.6–6.5); 4.2 mmol/L (3.7–5.0), respectively) for the lightweights and middleweights (*p* = 0.047, *p* = 0.004, respectively). Four horses (12%) had elevated urea levels (Normal laboratory range: 1.8–8.9 mmol/L) on arrival, reaching a maximum of 15.4 mmol/L.

As expected, there was a strong correlation between plasma total protein, albumin, globulin, creatinine and urea (Table 2).

Calculated osmolarity was overall greater on arrival (median: 267.0 mmol/Kg (258.3–278.5)) than pre- (median: 256.0 mmol/Kg (245.3–262.8) and post-competition (median: 250.5 mmol/Kg (241.5–259.5)) (*p* < 0.001). It decreased significantly in the middleweights between arrival (median: 281.0 mmol/Kg (256.5–295.0)) and post-competition (median: 242.0 mmol/Kg (241.0–247.5)) (*p* = 0.006) as well as between arrival (median: 267.0 mmol/Kg (264.3–267.8)) and pre-competition (median: 247.0 mmol/Kg (238.0–256.0)) in the heavyweights (*p* = 0.013).

Altogether, the increased values of total proteins, albumin, creatinine, urea and calculated osmolarity indicate that these horses were overall hemoconcentrated on arrival. However, the plasma chemistries of the heavyweight horses did not indicate hemoconcentration (except for higher albumin on arrival than pre-competition) after analysis by weight categories.

#### Electrolytes

Overall, sodium, chloride and potassium decreased after arrival. Sodium and chloride were significantly greater on arrival (median: 135.0 mmol/L (128.5–138.8); 99.0 mmol/L (96.0–103.0, respectively) than at both pre- (median: 129.5 mmol/L (123.3–131.0); 95.0 mmol/L (86.0–98.0), respectively) and post-competition (median: 125.5 mmol/L (122.0–131.5); 92.0 mmol/L (82.8–96.0), respectively) (*p* < 0.001, *p* = 0.004, respectively) (Table 1), and potassium was greater on arrival (median: 3.4 mmol/L (3.2–3.7)) than post-competition (median: 2.9 mmol/L (2.4–3.4)) (*p* = 0.034). Sodium, chloride and potassium decreased significantly between arrival (median: 140.0 mmol/L (127.5–131.8); 102.0 mmol/L (95.5–105.3); 3.3 mmol/L (3.2–3.5), respectively) and post-competition (median: 123.0 mmol/L (120.5–131.8); 91.0 mmol/L (82.3–94.3); 2.8 mmol/L (2.2–2.8), respectively) for the middleweight horses (*p* = 0.02, *p* = 0.01 and *p* = 0.01 respectively), while only sodium showed a decrease between arrival (median: 134.0 mmol/L (133.0–135.0)) and pre-competition (median: 125.0 mmol/L (119.5–129.5)) in the heavyweights (*p* = 0.02). None of the 3 electrolytes showed any change over time in the lightweight horses.

Arrival samples indicated that 7 (21%) and 4 (12%) of the 34 horses had greater than normal sodium and chloride levels, respectively. The highest chloride and sodium levels on arrival were 112.0 mmol/L and 148.0 mmol/L respectively, in a lightweight horse.

Plasma sodium was strongly correlated to total protein, albumin, and urea when all horses were included in the analysis (Table 2). Plasma chloride was strongly correlated to total protein (*r* = 0.92, *p* = 0.003), globulin (*r* = 0.93, *p* = 0.001) and sodium (*r* = 0.80, *p* = 0.02) only in the lightweight horses.

#### Muscle enzymes

Overall there was a significant increase in CK between arrival (median: 175.5 IU/L (150.3–253.0)) and post-competition (median: 225.5 IU/L (142.0–887.5)) (*p* = 0.04), though this was not observed within the individual weight categories. Eleven horses had elevated AST (laboratory range: 180-570 IU/L) and/or CK values (laboratory range: 110-700 IU/L) prior to competition (10 horses (30%) at arrival, 9 (35%) at pre-competition). Eight horses (40%) had elevated AST and/or CK post-competition, only one of which was not elevated pre-competition.

There was as strong correlation between AST and ALT, as well as between CK and ALT (Table 2).

#### Cardiac troponin hscTnT

High sensitivity cTnT stayed within normal range for all horses and the trend to increase after competition was not significant (Table 1).

#### Other chemistry parameters

Plasma glucose was greater on arrival (median: 5.9 mmol/L (5.4–6.6)) than pre-competition (median: 5.4 mmol/L (5.0–5.6)) overall (*p* = 0.026) as well as for the middleweight (median: 6.4 mmol/L (5.9–7.2); 5.4 mmol/L (5.2–5.8), respectively) (*p* = 0.047) and heavyweight (median: 5.8 mmol/L (5.3–6.1); 5.0 mmol/L (4.8–5.5), respectively) (*p* = 0.044) categories (Table 1).

Glucose was strongly correlated with creatinine, urea, total protein, albumin and globulin (Table 2).

Plasma total bilirubin decreased overall between arrival (median: 26.4 μmol/L (19.9–31.3)) and pre-competition (median: 17.7 μmol/L (14.9–21.8)) (*p* = 0.01), which was also observed for the lightweights (median: 25.0 μmol/L (16.4–30.0); 15.0 μmol/L (12.9–18.8), respectively) and middleweights (median: 48.3 μmol/L (28.7–62.3); 18.7 μmol/L (17.2–37.4), respectively) (*p* = 0.01, p = 0.02, respectively).

Total bilirubin was strongly correlated to creatinine, urea, albumin and glucose (Table 2).

## Discussion

Our study describes the physiological demands of the Heavy Horse Pull, a unique high-intensity, short duration discipline, where horses pull more than 2.5 times their weight for a distance of 14 ft. This is also the first descriptive analysis of “shrinkage” or rapid weight change in horses, with up to an average change in weight of 8.6%. However, this weight change did not seem to affect performance of the horses, likely because they had 34 to 82 h to recover prior to competition.

### Weight change and hemoconcentration

While some human athletes subject themselves to RWL to “make weight” in an attempt to gain a competitive edge, evidence supporting this claim of enhanced performance is disputed. It has been shown that in humans even a 2% change in body weight negatively affects aerobic and endurance performance [2]. It is believed that weight loss through dehydration affects sprint exercises less than endurance events [4, 14], and there is debate as to whether mild dehydration may actually improve performance in sprint and power sports in humans [15]. Although laboratory tests have shown reduced human athletic capacity following 2% weight loss, up to a 4% weight loss has not been shown to affect performance in field tests [16, 17].

There is, however, an important difference in rapid weight change between human athletes and draft horses entering pulling competitions: whereas human athletes compete at their lower-than-normal weight, heavy horses have a period of time regain significant weight before competing. Therefore, although horses may arrive to the event in a dehydrated state, we found that they compete in a euhydrated state. Indeed, in the present study, no horse had plasma creatinine or urea values above normal range at the pre-competition time point, and the calculated osmolarity, creatinine, urea and total protein values decreased significantly between arrival and pre-competition (Table 1). The rapid weight change may therefore have less of an effect on the performance and physiological parameters of these horses than on human athletes.

Work previously conducted in a different type of pulling event in Europe (light horses pulling for 1.3 min) where horses compete in a dehydrated and unfed state found that regardless of weight category, dehydrated horses had increased total protein, albumin, creatinine, sodium and urea [18]. Interestingly in the present study, not all horses that experienced weight loss were hemoconcentrated on arrival. This indicates that while most horses lost weight via a loss of intravascular fluid, some horses instead lost gastrointestinal content, possibly due to feed restriction or laxative use.

Lightweight category horses had the least amount of time to recover between arrival and competition (~34 h) compared to middleweights (~58 h) and heavyweights (~82 h). Based on this minimum period of 34 h, it does not appear that number of days before competition had a significant effect on pre-competition volemic status. Indeed, no horse had increased creatinine or urea at pre-competition, indicating that these horses were able to adequately rehydrate during the 34-h period.

Mild to moderate hemoconcentration (elevated total protein) was observed in 11 horses post-competition, which is a normal response following maximal exercise.

### Weight change and overall rank

The strong negative correlation (*r* = 0.69, *p* = 0.002) found between the weight change and overall ranking in middleweight horses (Fig. 3) indicates that the more weight the horse gained (indicative more intensive rapid weight change), the better they placed. However, it may also be that the best teams are also the ones that practice shrinkage. A study conducted on work oxen found that weight loss had no effect on pulling performance, however this was achieved through long-term diet restrictions with adequate water [19]. In the present study, horses that showed severe hemoconcentration did rehydrate quickly, and this rapid weight change did not impair performance (Fig. 3). Additionally, in spite of having the least amount of time (34 h) to replenish any weight loss, the lightweight horses pulled the greatest weight relative to their bodyweight, indicating that the rapid weight change is unlikely to have an effect on pulling performance as long as the horses have at least 34 h to recover.

Further research is required to determine if there is a physiological relationship between weight loss and rank at pulling competitions.

### Percentage of weight pulled

Draft capacity is often represented in terms of horsepower (the rate at which work is performed) with horses having been selected for their greater capacity and speed than oxen or cattle, indicating their superior horsepower [20]. Horses working in teams will have a greater cumulative draft capacity, however each individual horses’ draft capacity will be reduced by up to 7.5% for two horses when compared to a single animal effort [21].

Previously, research has focused on sustained pulling capacity of horses for farming where they pull 10–12% of their body weight [20]. In comparison, we found that these horses could pull over 2.5 times their body weight, though it was over a very short duration.

The competitors perceive that heavier horses have an advantage in the pulling competitions. In that sense, there was a trend in the present study to have a greater maximum weight pulled by the heavyweight teams. Combined data from this study and the historical results available from the Calgary Stampede pulling competitions over the previous 13 years, however, indicate that the maximum weight pulled does not differ between different weight classes. This suggests that the weight of the horses is not the most determining factor for the maximum pulling weight capacity of the teams during competition. It has been reported that small horses are stronger relative to their body weight compared to larger horses [22], with ponies able to pull 66% of the weight that was pulled by horses and 116% of that pulled by cattle [23]. Similarly, we found that lightweight horses pulled the greatest amount of weight relative to body mass (median: 275% (272–286)), which is greater than what a previous study reported on Valencian horses [18]. The Valencian horses described by Munoz et al., pulled 2–2.5 times their bodyweight for 1.3 min during competitions which is also much shorter than the draft horses pulling competition described in the present study,

### Plasma electrolytes and other chemistry parameters

There were many differences in plasma parameter levels between arrival and pre- or post-competition, as discussed below; however, no differences were found between pre- and post- competition. These post-competition values indicate that the duration of the event was simply too short to induce significant changes in the variables measured, or that the horses were able to recover from the competition within 3 h. Indeed, a different study found that recovery from pulling exercise happens quickly with a return of blood parameters to baseline levels within 15 to 30 min [18].

On arrival, plasma glucose was significantly greater than at pre-competition and there was a strong correlation between glucose and creatinine, urea, total protein, albumin and globulin. Altogether, these results are likely explained by the stress of shipping (some teams were shipped for longer than 17 h) combined with hemoconcentration.

Sodium and chloride significantly decreased between arrival and pre-competition and then post-competition. It is interesting to note that 9/34 horses on arrival were hyponatremic. The reason for this is unknown, but it is possibly due to sodium losses through the colon. In spite of these 9 horses being hyponatremic, the data shows overall greater plasma sodium levels on arrival than pre-competition. The higher overall plasma sodium and chloride levels on arrival were likely related to hemoconcentration due to shipping and rapid weight change practices. Similarly, a previous study found an increase in plasma sodium and chloride in dehydrated pulling horses compared to euhydrated controls, and found a similar correlation as we did between plasma sodium and urea [18].

In the study from Munoz et al. a pulling exercise induced a short duration increase in sodium and chloride that then decreased back to resting levels within 15 min in euhydrated horses [18]. In the present study however, we actually observed a trend for plasma sodium and chloride to decrease post-competition. This is probably because our post-competition samples were taken 3 h after exercise, when plasma sodium and chloride values were at a steady state and not affected by exercised-induced electrolyte shifts.

Surprisingly, plasma potassium decreased significantly between arrival and post-competition (Table 1). Potassium is usually related to hay intake – it is difficult to explain this finding in the context of RWL, where we would have expected lower potassium values on arrival due to feed withdrawal, and an increase in plasma potassium with time. A confounding factor may be hemoconcentration on arrival.

We found an increase in CK between arrival and post-competition, likely due to the effect of shipping, as CK takes a few hours to peak after muscle damage is initiated. In addition, 7/ 20 horses had increased CK values post-competition (median: 225.5 IU/L (142.0–887.5)). Since these samples were taken 3 h after the competition, we estimate that this is a good indication of the true CK peak values after the pulling event. The CK values noted were under 5000 IU/L for all except for 2 horses (8784 IU/L and 16,605 IU/L). This altogether with the fact that CK values did not increase significantly between pre- and post-competition, shows that the pulling event did not have an effect on the muscle metabolism of these draft horses. There were no significant changes in AST values, however AST peaks later than CK and a longer post-competition period would be required to observe this peak. Total bilirubin decreased significantly overall between arrival and pre-competition, which may be explained by a decreased feed intake right before and during shipping.

In the present study we found a trend for hscTnT to increase post-competition that was not statistically significant. This indicates that there is no myocardial damage induced by the high intensity short duration pulling exercise in draft horses.

## Conclusions

Our study showed that horses competing in the Heavy Horse Pull undergo significant weight changes, most marked in the middleweight category. Although we found that horses arrived to the event with some indications of hemoconcentration, the minimum 34 h delay before the competition allowed the horses to recover well from the effects of shipping and rapid weight change. We did not find any effect of the pulling competition on the chemistry parameters, indicating that these events are well tolerated and only induce reversible, short-term stress on the metabolism of the horses.

## Abbreviations

ALT: Alanine aminotransferase
AST: Aspartate aminotransferase
CK: Creatine kinase
cTnT: Cardiac troponin
hscTnT: High-sensitivity cardiac troponin
RWL: Rapid weight loss

## Footnotes

1 AU680, Beckman Coulter Inc., Mississauga, ON.

2 Troponin T hs, Roche Diagnostics, Indianapolis, IN.

3 Cobas-e601, Roche Diagnostics, Indianapolis, IN.

4 Calgary Lab Services, Calgary, AB.
